# TSAT: Efficient evaluation software for NGS data of phage/mirror-image phage display selections

**DOI:** 10.1016/j.bpr.2024.100166

**Published:** 2024-06-21

**Authors:** Tim Altendorf, Jeannine Mohrlüder, Dieter Willbold

**Affiliations:** 1 Institut für Biologische Informationsprozesse, IBI-7, Forschungszentrum Jülich, Jülich, Germany; 2 Institut für Physikalische Biologie, Heinrich-Heine-Universität Düsseldorf, Düsseldorf, Germany

**Keywords:** Phage display, Mirror-image phage display, antibodies, peptides, protein-protein interaction, evaluation software

## Abstract

Phage display and mirror-image phage display are commonly used techniques for the identification of binders that are specific to predefined targets. Recent studies demonstrated the effectiveness of next-generation sequencing (NGS) by increasing the amount of information extracted from selections. This allows for a better analysis and increases the possibility to select effective binders. A potential downside to NGS analysis of phage display selections is the increased workload that is needed to analyze the obtained information. Here, we report on the development of TSAT (target-specific analysis tool), software for user-friendly and efficient analysis of peptide sequence data from NGS of phage display selections.

## Why it matters

A distinct advantage of phage display and mirror-image phage display is the high variability of phages (10^9^) that can be screened at the same time. Traditional sequencing methods imposed a bottleneck on this variability, as only a small number of sequences could be processed at the same time. New techniques in the field of sequencing, e.g., next-generation sequencing (NGS), break these bottlenecks and allow for the sequencing of millions of phages at the same time. This in turn leads to a secondary bottleneck, as many evaluation systems are not equipped to process this increase in data. TSAT (target-specific analysis tool) aims to help with the identification and processing of nucleotide data obtained from NGS.

## Introduction

Classical phage display (1,2) or mirror-image phage display (3) selections may yield peptides, antibodies, or other biomolecules that are highly specific to a target after several rounds of selection. These techniques were used successfully in the identification of binders for potential disease-causing proteins such as Aβ (4) or certain types of cancer (5,6,7,8,9) and are widely used for antibody generation (10,11,12) and in different disciplines, such as material science (13). The number of selection rounds required may vary depending on the target used. In case of well-structured targets, the process may be faster than for targets that do not have a fixed three-dimensional structure. For these targets, it may take more effort to generate a high enrichment of binding phages, which is required to identify them using classical sequencing methods such as Sanger sequencing. Selections with many rounds of selection are inherently problematic because the diversity of phages decreases more than intended as the number of rounds increases (14). In addition, the increase in selection rounds leads to an enrichment of phages that have an advantage in the amplification that is performed after each round of selection (14). The effect of these problems can be lessened by using new sequencing methods such as next-generation sequencing (NGS), which dramatically increases the number of sequences that can be analyzed from a selection. This allows for better evaluation of binder quality. By comparing the different selection rounds with each other, it is possible to identify binders that are enriched in each selection round. This analysis can be improved by incorporating control selections, which greatly reduces the likelihood of selecting non-target-specific binders (15). Although NGS has all these improvements, it also brings its own challenges. The analysis of generated genetic information is often not established, and traditional software is often either unable to analyze these datasets or requires a long time. This is especially true for NGS data from phage display selections, as the end user is only interested in a specific part of the genome that encodes the randomized peptide, antibody, and so forth. A professional software solution or service that analyzes and provides the data to the end user is often a costly alternative. To solve this problem, we have developed the TSAT (target sequence analysis tool) program. TSAT is capable of analyzing up to three rounds of selection with control selections for a user-defined region of interest. It translates the DNA found into its corresponding amino acids and stores them in a database. It can also be used to perform additional filtering to increase the probability of finding specific peptide binders. We present TSAT as open-source software so that it can be used by everyone to identify target-specific binders for desired targets.

## Materials and methods

### Modules used during development

TSAT was developed using the modules Tkinter, Bio, base64, re, collections, sqlite3, time, datetime, sys, and threading, and was written in Python version 3.6.5. It is a stand-alone executable program.

### Processing of provided data

TSAT accepts data in the form of FASTQ files encoded with UTF-8. A provided file is divided into several sections starting with each new word. Within a section, TSAT searches for the first occurrence of the identifying information and extracts the corresponding peptide nucleotide information. Thereafter, the next section is evaluated.

### Identification of peptide nucleotide information

TSAT identifies desired nucleotide information via a regular expression function. This identification is dependent on framing regions surrounding the desired nucleotide information, as they are part of the search pattern given to the function. The information on the search pattern has to be provided but may be saved in a database accessible with SQL for fast accessibility during future reuses. The search pattern is expected in the format XXX(.+?)XXX, where (.+?) represents the unknown region of interest and XXX stands for the framing nucleotides, which have to be provided by the user. During the operation the framing regions denoted by XXX define when the desired information starts and when it ends. The (.+?) modifier defines that the program should match all characters that are between the two framing regions, then stop after the first match and return the result. This approach was used to increase the analysis speed of TSAT and to ensure that each genetic sequence can only provide one returned peptide. An example for a valid search pattern would be GATTCCAGG(.+?)TACGACCCG. All provided data will be searched with the same search pattern by the regular expression function; it is not possible to mix search patterns during an ongoing analysis.

### Processing of extracted nucleotide information

The extracted nucleotide data are checked as to whether it is possible to translate or if a deletion has occurred that would lead to a loss of reading frame. This is achieved by controlling whether the length of DNA extracted via the regular expression function is dividable by 3. Data that fail this check are registered in a separate list, and the user is informed about the frequency of incomplete sequences by TSAT. Data that passed this checkpoint are translated according to the user. Nucleotide information is counted on the DNA level, so multiple protein sequences with the same amino acid composition but different frequencies may be present. There are four different options that are selectable by the user. Forward translates the data directly into amino acids. Forward complement creates a complement strand of DNA and translates this newly synthesized strand into amino acids. Reverse generates a reverse strand of the DNA and translates it into amino acids. Reverse complement generates the reverse complement strand of DNA and translates this strand into amino acids. The translated data are stored in a database. Saved are the selection name, user, sequence, total frequency, and frequency adjusted to ppm as well as the date that the data were processed and stored.

### Filtering and ranking of obtained peptide sequences

TSAT is equipped with the ability to filter and order sequences according to predefined values, the so-called empty and enrichment score. This function requires control selections. Control selections are provided to the program in three different categories. A direct control describes a selection that was first made with a target and then continued in the next round without a target. The generated information is used to ensure the specificity of a sequence to the target. A target-specific sequence would not accumulate in a direct control selection in the same amount. Similarly, an empty selection describes a selection made entirely without the target. This is used to identify and eliminate potential nonspecific binders. The third control consists of the sequenced phage library before the selection was performed. This control allows the identification of sequences with a high prevalence within the phage library and is critical for generating the enrichment score. The empty and enrichment scores are calculated according to the following formulas:

$$\text{Enrichment}\;\text{score}=\frac{\text{frequency}\;\text{of}\;a\;\text{sequence}\;\text{in}\;\text{Target}\;\text{Selection}\;3}{\text{frequency}\;\text{of}\;\text{the}\;\text{same}\;\text{sequence}\;\text{in}\;\text{library}}\text{,}$$

$$\text{Empty}\;\text{score}=\frac{\text{frequency}\;\text{of}\;a\;\text{sequence}\;\text{in}\;\text{Target}\;\text{Selection}\;3}{\text{frequency}\;\text{of}\;\text{the}\;\text{same}\;\text{sequence}\;\text{in}\;\text{Empty}\;\text{Selection}\;3}\text{.}$$

Data are ordered according to the empty score. All calculations and filtering are performed with normalized values to ensure comparison without bias.

### Long-term storage of processed data

As previously stated, evaluated as well as potential filtered data are stored in a database for long-term storage. This is achieved by transferring the data to a given database via SQL commands. The user is able to select the location in which the database will be created, allowing for storage on a local computer or within a network. The data are accessible and editable using SQL commands. After a complete analysis using TSAT, the given database includes a table for every provided selection round (target selections, control selections, empty selections, library). Assuming that the user has performed the filtering, an additional evaluation table is created where the filtered results are stored. This design keeps the original data that have been extracted from the selection rounds in case the evaluation yields unexpected results. To directly view data in the database, external software is needed.

## Results

TSAT is able to effectively analyze NGS analysis with millions of reads. The average time it takes for TSAT to complete a full analysis depends on the provided file size and the hardware used. From our experiences, it took TSAT around 30 min to analyze a full selection with all controls where each file contained around one million reads.

## Discussion

Using TSAT, we were able to efficiently evaluate the results of several complex NGS runs from mirror-image phage display selections. These selections targeted proteins for which traditional sequencing methods did not yield satisfactory binders. With NGS and TSAT, we were able to achieve much higher resolution of selection results. The additional benefit of control selection analysis and filtering enabled rapid and efficient identification of potential binding partners. TSAT also enabled us to identify and reduce sequences that are not target specific and are present due to either amplification advantages or binding to other surfaces, such as plastic binders. This was done by comparing selections against different targets and identifying sequences that were enriched in all of them. Selections against human superoxide dismutase 1 (15), polyglutamine (16), microtubule-associated protein tau (17), and SARS-CoV-2 (18) were all analyzed using TSAT. Other not yet published targets include, for example, *α*-synuclein. A potential problem encountered during testing was that TSAT cannot account for data with a point mutation in the framing regions. It is possible to analyze these “missed” sequences by changing the framing regions for a particular point mutation. When we searched the datasets we selected using this method, we found that the overall distribution of sequences was very similar to the sequences without point mutation in the framing regions. This problem could be addressed in a later version by changing the process by which TSAT handles the detection of framing regions.

### Summary

TSAT is open-source software that can be used to effectively and efficiently analyze NGS results from phage display and mirror-image phage display selections. It has been used to identify binders against multiple and different protein targets. It enables the identification of sequences that are unlikely to be associated with the target and in the identification of target-specific sequences that would otherwise be missed. Because it is open-source software, it can be used by everyone free of charge.

Zenodo DOI: https://zenodo.org/doi/10.5281/zenodo.10342034. GitHub: https://github.com/taltendorf/Target-Sequence-Analysis-Tool-TSAT-.

## Author contributions

T.A. wrote the manuscript and developed the software. D.W. and J.M. supervised the project and revised the manuscript. J.M. tested the software.

## Supporting material

Document S1. User guide

Document S2. Article plus supporting material
