# Supplementary material for: TSAT: Efficient evaluation software for NGS data of phage/mirror-image phage display selections
Source: Biophys Rep (N Y). 2024 Jun 21;4(3):100166. doi: 10.1016/j.bpr.2024.100166 (PMC11269273; doi:10.1016/j.bpr.2024.100166)
Supplement: Document S1. User guide [file mmc1.pdf]

Biophysical Reports, Volume *4*

Supplemental information

TSAT: Efficient evaluation software for NGS data of phage/mirror-image phage display selections

Tim Altendorf, Jeannine Mohrlüder, and Dieter Willbold

Target Sequence Analysis Tool (TSAT)

User Guide

Contents

Source code & Software 2
Objective of TSAT 2
How to use 3
Startup 3
Database creation 3
Translation direction 4
Framing region input 4
Input user and selection 7
Data insertion 7
Start data extraction and translation 8
Filtering of processed data 8
Q&A 9

Source code & Software

The Source code for TSAT can be found on Github:

https://github.com/taltendorf/Target-Sequence-Analysis-Tool-TSAT-

Alternatively, the source code can be found under the following DOI:

https://zenodo.org/doi/10.5281/zenodo.10342034

A released executable version of TSAT can be downloaded here:

https://github.com/taltendorf/Target-Sequence-Analysis-Tool-TSAT-/releases/tag/v1.0

Objective of TSAT

TSAT is a computational tool for the rapid analysis of NGS data obtained from Phage display and Mirror Image Phage display selections. It is able to extract desired genetic information, translate the information from DNA into protein and in an optional second step filter the obtained information for sequences with a higher probability to bind targets while excluding sequences that may not be target specific due to unspecific binding, amplification advantages or a bias in the library distribution. It was developed using the modules Tkinter, Bio, base64, re, collections, sqlite3, time, datetime, sys, threading and was written in Python version 3.6.5.

Prerequisite

TSAT accepts data in the form of FASTQ files encoded with UTF-8. To directly investigate the processed information a secondary program to visualize databases will be needed (e.g., db-browser for sqlite). Extraction of sequence information and translation into protein can be done without secondary programms. For the filtering of unwanted sequences as well as generation of different scores TSAT needs at least one library file, one target selection as well as one empty selection of the same round as input data.

How to use

Upon start TSAT needs information input from the user to function correctly. Following is a step-by-step guide on how to fill in all the information.

Startup

TSAT can be started by running the executable or source code with a python interpreter. After successful startup the user is able to see the following Window.

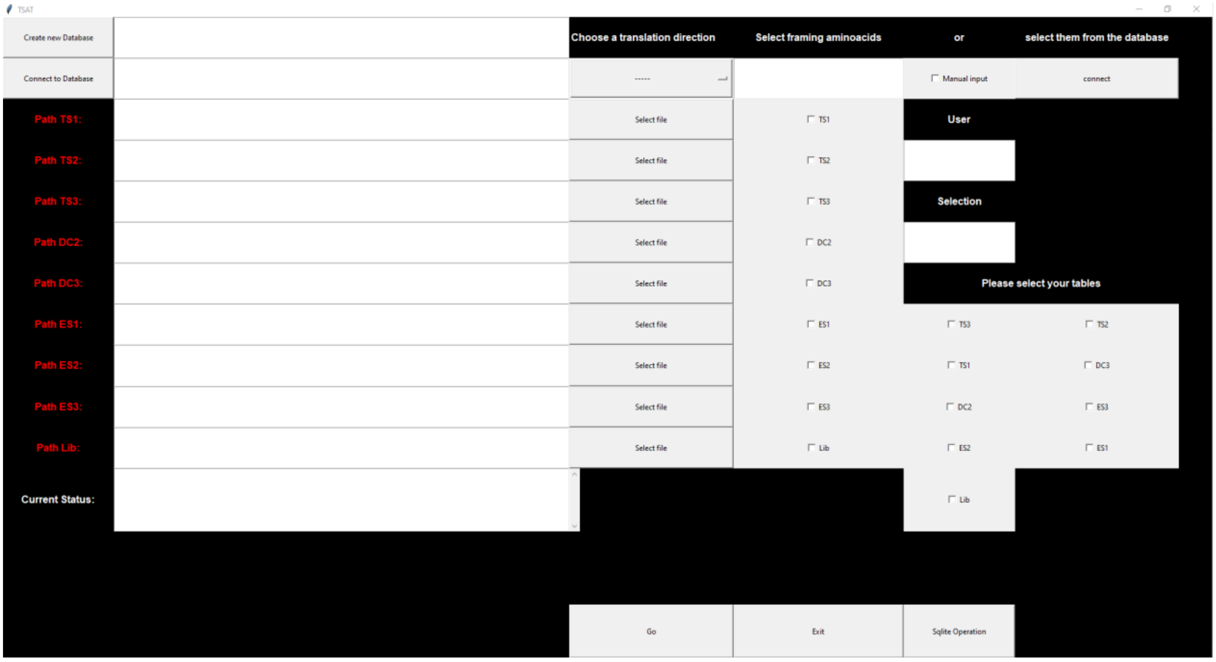

Database creation

In a first step the user needs to create a new database or connect to an existing one. It is recommended to create a new database for every run of TSAT to avoid loss or corruption of data. By clicking the “create new database” button the user is able to create and name a new database file (.db). Alternatively, the user can connect to an already existing database with the “Connect to Database” button.

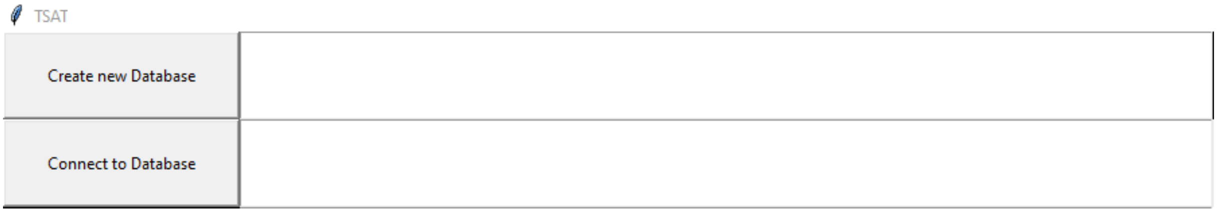

After creation of a new database, it is important to connect to the new database.

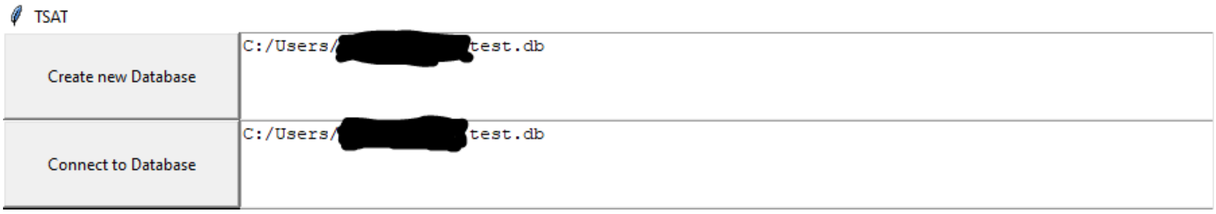

Translation direction

Here the user has to choose one of four possibilities for how the translation of excised DNA should be performed. Depending on how the phage library is set up the possibilities are Forward, Reverse, Forward complement and Reverse complement. This is done by choosing one of for options under the "Choose a translation direction" button. For the test data set the forward direction should be used.

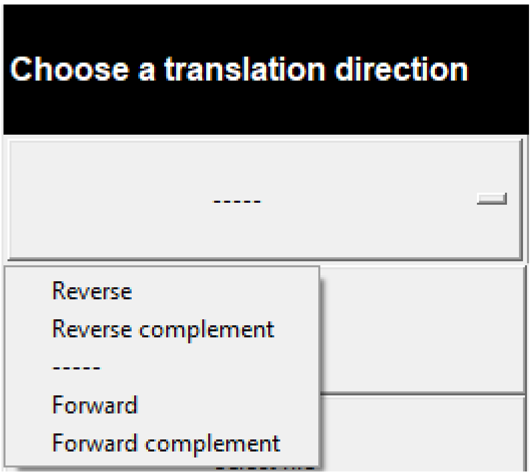

Framing region input

This step is of utmost importance as it provides TSAT with the information which random region is the region of interest .TSAT identifies desired nucleotide information via a regular expression function. This identification is dependent on framing regions surrounding the desired nucleotide information as they are part of the search pattern given to the function. For this you have two possibilities. The first one is to manually input the required information into TSAT. This option can be useful if you have a dataset that you only need to process once. For this you have to check the box "Manual Input" and insert the framing regions into the text box under "Select framing amino acids". Note that you have to give a defined amino acid composition before the randomized region and after it. The randomized region is displayed by (.+?)

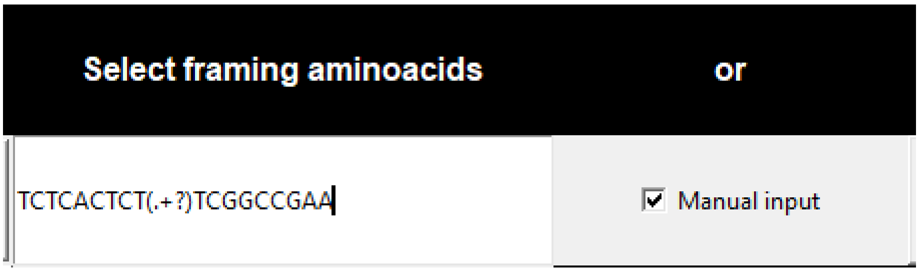

Alternatively, you can save your framing regions in a database and connect TSAT to this database. This is especially useful if you have many selections with the same randomized region, as it allows you to input the framing regions in a reduced amount of time with no spelling errors. In order to use this feature, you need to create a fresh database. This can be done clicking the “Create new Database” button in the top left corner of TSAT. After that you can connect to this database via the “Connect” button in the top right corner of TSAT. If you created a new database without any framing regions and connected to it two new buttons will appear below the “Connect” button.

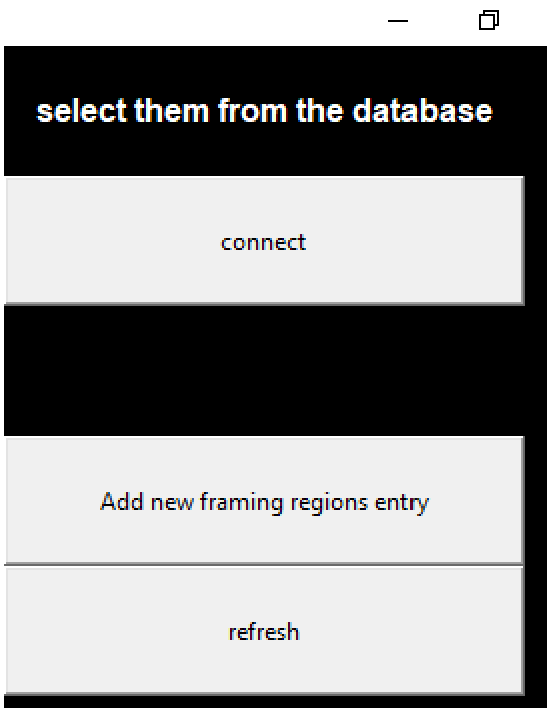

The next step is to add the wanted framing regions into the newly created database. This is done by clicking the button “Add new framing regions entry”. By clicking this button, a new window will open where you can insert the needed Information.

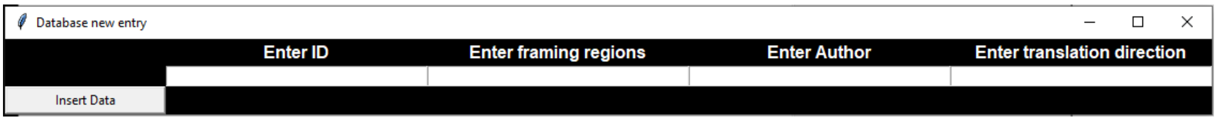

Here you need to give this framing region entry a name. This name will later be displayed in TSAT so that you can differentiate between different framing regions. Under framing regions, you have to enter the information that TSAT needs to find the desired randomized region. This is the same information you would have to enter in the manual mode. For this example, the framing regions that will be added are TCTCACTCT(.+?)TCGGCCGAA with (.+?) standing for the unknown randomized region. Afterwards you can add an author name which will only be saved in the database and not be displayed. Finally, you can enter a translation direction. At this moment the entry is also only saved in the database. It does not replace the drop-down selection within the main TSAT software. With the “Insert Data” button the

information is transferred to the database and saved within. Be sure that you have inserted everything correctly as you cannot change the data with TSAT. After this you can use the “refresh” button to update you connection with the Database. A new dropdown menu should appear in which you can select the entry you just created.

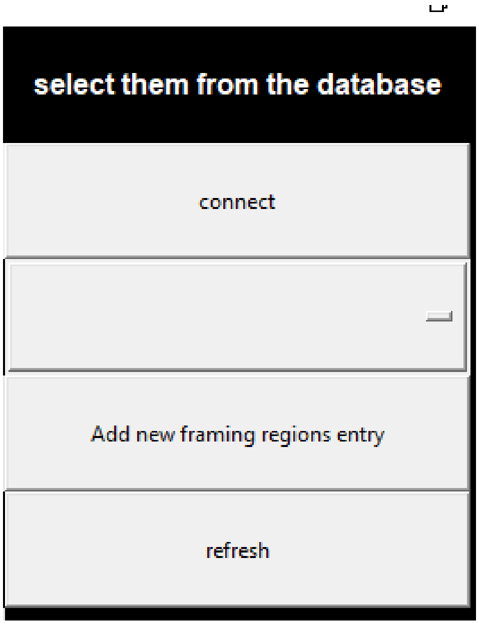

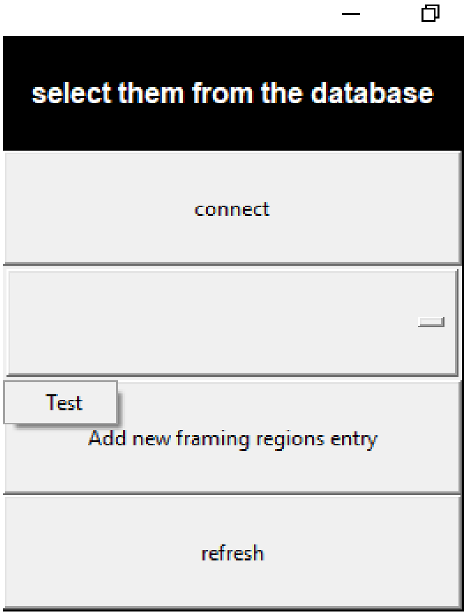

A test database containing the framing regions for the example dataset is provided on TSAT’s Github-page in the test folder. It is named framing regions.

Input user and selection

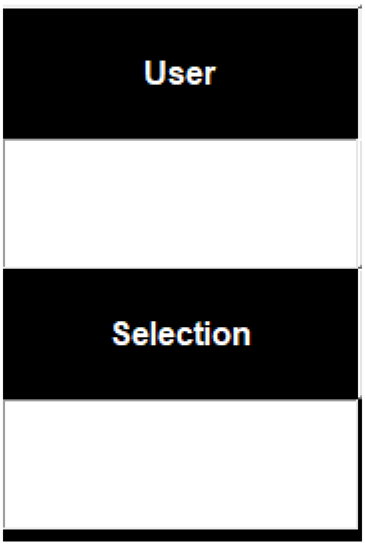

Here you can insert a user and a name for the selection. Both will be saved in the database. This is optional and TSAT will leave these areas blank if no entry was given.

Data insertion

The data that TSAT will analyze must be provided by the user using the “select file” button beside the textbox of “Path TS1” to “Lib”. The user is asked to provide a FASTQ file coded in UTF-8. Only one file can be given per path.

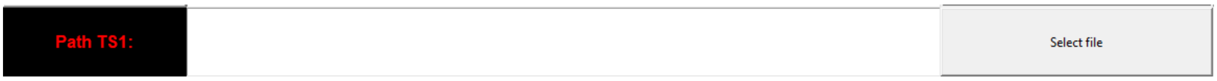

If the user wants to use the optional filtering it is important that the corresponding file is inserted into each path. TS stands for Target selection. This selection should be done against the target and is divided into 1-3 according to the increasing selection rounds. ES stands for empty selection. This selection is not against the target and should be done in parallel to the Target selection. It is used to identify sequences that are unlikely to be associated with the target but are present due unspecific binding. Again, this is divided into 1-3 according to the selection round. DC stands for Direct control. This selection was conducted against the target in the previous round and is subsequently performed without a target. DC selections are divided into 2 and 3 as the first round has to be the TS1 round. The library file is the final path option and represents the phage library state before a selection was performed.

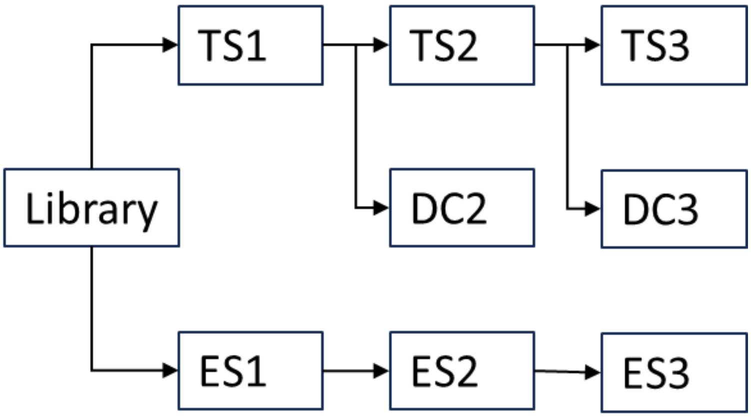

This file is used to identify sequences that are elevated in their frequency due to an amplification advantage or bias of the library. After file selection the path to the file is presented in the window. In case of a mix up it is possible to change the selected file by selecting another. After the files have been selected the user has to check which files were provided by marking the checkboxes to the right of the select file buttons.

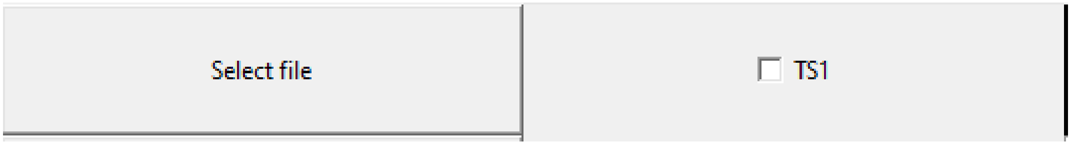

This is vital as TSAT won't operate on files that were not confirmed here. A user can use this feature to include or exclude a certain selection round for a second analysis without having to re-enter general Information. If a second analysis is performed, it is recommended to create a new database and connect to this newly created database. TSAT does not overwrite information already present in the database.

Start data extraction and translation

After all the required information has been entered and selected the operation may be started by using the "Go" button at the bottom of the program. TSAT will analyze the provided information and provide an overview over the progress in the current status box. An example of the displayed information would be the number of sequences that were found in a certain file. The textbox will also display the information when the processing has finished.

Filtering of processed data

The second functionality of TSAT is to filter the processed data in order to identify sequences that have a higher probability of being target specific and to exclude sequences that are thought to have a high frequency due to amplification advantage, starting bias or non-specific binding. To use this feature, one can either connect to a database containing at least one target selection, one empty selection and a library file or directly use the feature after finishing an extraction a translation with at least the mentioned files. To start the filtering, check the boxes corresponding to the files that are contained in the connected database under "please select your tables". The filtering is started by pressing the "Sqlite Operation" button at the bottom of TSAT. The filtering may take a while depending on the operating system. During the filtering TSAT will create two new scores to present two key features of every

sequence, the empty and enrichment score. The enrichment score represents the frequency of a sequence during its last target selection round divided by the frequency of the same sequence in the library.

$$Enrichment\ score = \frac{frequency\ of\ a\ sequence\ in\ TS3}{frequency\ of\ the\ same\ sequence\ in\ library}$$

This score is used to identify sequences that are only present as a result of a biased sequence distribution within the used phage library. The empty score represents the frequency of a sequence during its last target selection round divided by the frequency of the same sequence in the last empty selection round.

$$Empty\ score = \frac{frequency\ of\ a\ sequence\ in\ TS3}{frequency\ of\ the\ same\ sequence\ in\ ES3}$$

This score is used to identify sequences that are present as a result of binding to other components of the selection setup (e.g., plate, selection medium or blocking agent). TSAT excludes sequences that are smaller than 8 amino acids. Additionally, every sequence is analyzed for its enrichment from target round to target round as well as compared to the empty selection and direct control.

$$TS3 \geq ES3\ \&\ TS3 \geq DC3\ \&\ TS3 \geq TS2\ \&\ TS2 \geq ES2\ \&\ TS2 \geq DC2\ \&\ TS2 \geq TS1\ \&\ TS1 \geq ES1\&\ TS1 \geq Library$$

Sequences that do not increase from one target round to the next are excluded. Sequences that have a higher frequency in the corresponding empty selection and sequences whose frequency increases in a direct control compared to the previous target selection are removed. A new file is created after the filtering is completed. The user can decide where the file should be saved and might rename it. The file will be saved as a .fasta file. All sequences that were not discarded are saved in FASTA format. Sequences are sorted in descending order according to their respective Empty score. The empty score will be displayed in the .fasta file. In order to compare the enrichment scores a software to visualize the contents of a SQlite database is needed.

Q&A

Q: I see a star shape in my protein code, what is that?

A: The star shape is used if a stop codon was translated. This might happen when you use strains that substitute a certain stop codon with another amino Acid (e.g., amber suppressor).

Q: How can I review the translated data that is stored in the database?

A: TSAT cannot display the translated data that is stored in the database. For this please use a different software that is able access and display such data. As an example, you could use DB browser for SQLite

Q: How does TSAT handle sequences that are not present in the empty selection or library?

A: In this case the sequence will be handled as if it was present 0.5 times in the selection or library.

Q: Why do I need the library file for filtering?

A: The library is used to check the state before a selection occurred. In the filtering it serves as a "target selection 0".

Q: Why do I see the same sequence twice or more often?

A: This happens when there are multiple codons that code for the same amino acid. TSAT orders at the level of DNA meaning those "duplicated" sequences all have a different genetic code.
