# Supplementary material for: TSAT: Efficient evaluation software for NGS data of phage/mirror-image phage display selections
Source: Biophys Rep (N Y). 2024 Jun 21;4(3):100166. doi: 10.1016/j.bpr.2024.100166 (PMC11269273; doi:10.1016/j.bpr.2024.100166)
Supplement: Document S2. Article plus supporting material [file mmc2.pdf]

Biophysical Reports

REPORT

TSAT: Efficient evaluation software for NGS data of phage/mirror-image phage display selections

Tim Altendorf,[1,2] Jeannine Mohrlüder,[1,*] and Dieter Willbold[1,2,*]
[1]Institut für Biologische Informationsprozesse, IBI-7, Forschungszentrum Jülich, Jülich, Germany and [2]Institut für Physikalische Biologie, Heinrich-Heine-Universität Düsseldorf, Düsseldorf, Germany

ABSTRACT Phage display and mirror-image phage display are commonly used techniques for the identification of binders that are specific to predefined targets. Recent studies demonstrated the effectiveness of next-generation sequencing (NGS) by increasing the amount of information extracted from selections. This allows for a better analysis and increases the possibility to select effective binders. A potential downside to NGS analysis of phage display selections is the increased workload that is needed to analyze the obtained information. Here, we report on the development of TSAT (target-specific analysis tool), software for user-friendly and efficient analysis of peptide sequence data from NGS of phage display selections.

WHY IT MATTERS A distinct advantage of phage display and mirror-image phage display is the high variability of phages (10^9) that can be screened at the same time. Traditional sequencing methods imposed a bottleneck on this variability, as only a small number of sequences could be processed at the same time. New techniques in the field of sequencing, e.g., next-generation sequencing (NGS), break these bottlenecks and allow for the sequencing of millions of phages at the same time. This in turn leads to a secondary bottleneck, as many evaluation systems are not equipped to process this increase in data. TSAT (target-specific analysis tool) aims to help with the identification and processing of nucleotide data obtained from NGS.

INTRODUCTION

Classical phage display (1,2) or mirror-image phage display (3) selections may yield peptides, antibodies, or other biomolecules that are highly specific to a target after several rounds of selection. These techniques were used successfully in the identification of binders for potential disease-causing proteins such as Aβ (4) or certain types of cancer (5–9) and are widely used for antibody generation (10–12) and in different disciplines, such as material science (13). The number of selection rounds required may vary depending on the target used. In case of well-structured targets, the process may be faster than for targets that do not have a fixed three-dimensional structure. For these targets, it may take more effort to generate a high enrichment of binding phages, which is required to identify them using classical sequencing methods such as Sanger sequencing. Selections with many rounds of selection are inherently problematic because the diversity of phages decreases more than intended as the number of rounds increases (14). In addition, the increase in selection rounds leads to an enrichment of phages that have an advantage in the amplification that is performed after each round of selection (14). The effect of these problems can be lessened by using new sequencing methods such as next-generation sequencing (NGS), which dramatically increases the number of sequences that can be analyzed from a selection. This allows for better evaluation of binder quality. By comparing the different selection rounds with each other, it is possible to identify binders that are enriched in each selection round. This analysis can be improved by incorporating control selections, which greatly reduces the likelihood of selecting non-target-specific binders (15). Although NGS has all these improvements, it also brings its own challenges. The analysis of generated genetic information is often not established, and traditional software is often either unable to analyze these datasets or requires a long time. This is especially true for NGS

Submitted January 30, 2024, and accepted for publication June 20, 2024.
*Correspondence: j.mohrlueder@fz-juelich.de or d.willbold@fz-juelich.de
Editor: Jorg Enderlein.
https://doi.org/10.1016/j.bpr.2024.100166
© 2024 The Authors. Published by Elsevier Inc. on behalf of Biophysical Society.
This is an open access article under the CC BY license (http://creativecommons.org/licenses/by/4.0/).

data from phage display selections, as the end user is only interested in a specific part of the genome that encodes the randomized peptide, antibody, and so forth. A professional software solution or service that analyzes and provides the data to the end user is often a costly alternative. To solve this problem, we have developed the TSAT (target sequence analysis tool) program. TSAT is capable of analyzing up to three rounds of selection with control selections for a user-defined region of interest. It translates the DNA found into its corresponding amino acids and stores them in a database. It can also be used to perform additional filtering to increase the probability of finding specific peptide binders. We present TSAT as open-source software so that it can be used by everyone to identify target-specific binders for desired targets.

MATERIALS AND METHODS

Modules used during development

TSAT was developed using the modules Tkinter, Bio, base64, re, collections, sqlite3, time, datetime, sys, and threading, and was written in Python version 3.6.5. It is a stand-alone executable program.

Processing of provided data

TSAT accepts data in the form of FASTQ files encoded with UTF-8. A provided file is divided into several sections starting with each new word. Within a section, TSAT searches for the first occurrence of the identifying information and extracts the corresponding peptide nucleotide information. Thereafter, the next section is evaluated.

Identification of peptide nucleotide information

TSAT identifies desired nucleotide information via a regular expression function. This identification is dependent on framing regions surrounding the desired nucleotide information, as they are part of the search pattern given to the function. The information on the search pattern has to be provided but may be saved in a database accessible with SQL for fast accessibility during future reuses. The search pattern is expected in the format XXX(.+?)XXX, where (.+?) represents the unknown region of interest and XXX stands for the framing nucleotides, which have to be provided by the user. During the operation the framing regions denoted by XXX define when the desired information starts and when it ends. The (.+?) modifier defines that the program should match all characters that are between the two framing regions, then stop after the first match and return the result. This approach was used to increase the analysis speed of TSAT and to ensure that each genetic sequence can only provide one returned peptide. An example for a valid search pattern would be GATTCCAGG(.+?)TACGACCCG. All provided data will be searched with the same search pattern by the regular expression function; it is not possible to mix search patterns during an ongoing analysis.

Processing of extracted nucleotide information

The extracted nucleotide data are checked as to whether it is possible to translate or if a deletion has occurred that would lead to a loss of reading frame. This is achieved by controlling whether the length of DNA extracted via the regular expression function is dividable by 3. Data that fail this check are registered in a separate list, and the user is informed about the frequency of incomplete sequences by TSAT. Data that passed this checkpoint are translated according to the user. Nucleotide information is counted on the DNA level, so multiple protein sequences with the same amino acid composition but different frequencies may be present. There are four different options that are selectable by the user. Forward translates the data directly into amino acids. Forward complement creates a complement strand of DNA and translates this newly synthesized strand into amino acids. Reverse generates a reverse strand of the DNA and translates it into amino acids. Reverse complement generates the reverse complement strand of DNA and translates this strand into amino acids. The translated data are stored in a database. Saved are the selection name, user, sequence, total frequency, and frequency adjusted to ppm as well as the date that the data were processed and stored.

Filtering and ranking of obtained peptide sequences

TSAT is equipped with the ability to filter and order sequences according to predefined values, the so-called empty and enrichment score. This function requires control selections. Control selections are provided to the program in three different categories. A direct control describes a selection that was first made with a target and then continued in the next round without a target. The generated information is used to ensure the specificity of a sequence to the target. A target-specific sequence would not accumulate in a direct control selection in the same amount. Similarly, an empty selection describes a selection made entirely without the target. This is used to identify and eliminate potential nonspecific binders. The third control consists of the sequenced phage library before the selection was performed. This control allows the identification of sequences with a high prevalence within the phage library and is critical for generating the enrichment score. The empty and enrichment scores are calculated according to the following formulas:

$$\text{Enrichment score} = \frac{\text{frequency of a sequence in Target Selection 3}}{\text{frequency of the same sequence in library}},$$

$$\text{Empty score} = \frac{\text{frequency of a sequence in Target Selection 3}}{\text{frequency of the same sequence in Empty Selection 3}}.$$

Data are ordered according to the empty score. All calculations and filtering are performed with normalized values to ensure comparison without bias.

Long-term storage of processed data

As previously stated, evaluated as well as potential filtered data are stored in a database for long-term storage. This is achieved by transferring the data to a given database via SQL commands. The user is able to select the location in which the database will be created, allowing for storage on a local computer or within a network. The data are accessible and editable using SQL commands. After a complete analysis using TSAT, the given database includes a table for every provided selection round (target selections, control selections, empty selections, library). Assuming that the user has performed the filtering, an additional evaluation table is created where the filtered results are stored. This design keeps the original data that have been extracted from the selection rounds in case the evaluation yields unexpected results. To directly view data in the database, external software is needed.

RESULTS

TSAT is able to effectively analyze NGS analysis with millions of reads. The average time it takes for TSAT to complete a full analysis depends on the provided file size and the hardware used. From our experiences, it took TSAT around 30 min to analyze a full selection with all controls where each file contained around one million reads.

DISCUSSION

Using TSAT, we were able to efficiently evaluate the results of several complex NGS runs from mirror-image phage display selections. These selections targeted proteins for which traditional sequencing methods did not yield satisfactory binders. With NGS and TSAT, we were able to achieve much higher resolution of selection results. The additional benefit of control selection analysis and filtering enabled rapid and efficient identification of potential binding partners. TSAT also enabled us to identify and reduce sequences that are not target specific and are present due to either amplification advantages or binding to other surfaces, such as plastic binders. This was done by comparing selections against different targets and identifying sequences that were enriched in all of them. Selections against human superoxide dismutase 1 (15), polyglutamine (16), microtubule-associated protein tau (17), and SARS-CoV-2 (18) were all analyzed using TSAT. Other not yet published targets include, for example, α-synuclein. A potential problem encountered during testing was that TSAT cannot account for data with a point mutation in the framing regions. It is possible to analyze these "missed" sequences by changing the framing regions for a particular point mutation. When we searched the datasets we selected using this method, we found that the overall distribution of sequences was very similar to the sequences without point mutation in the framing regions. This problem could be addressed in a later version by changing the process by which TSAT handles the detection of framing regions.

Summary

TSAT is open-source software that can be used to effectively and efficiently analyze NGS results from phage display and mirror-image phage display selections. It has been used to identify binders against multiple and different protein targets. It enables the identification of sequences that are unlikely to be associated with the target and in the identification of target-specific sequences that would otherwise be missed. Because it is open-source software, it can be used by everyone free of charge.

Zenodo DOI: https://zenodo.org/doi/10.5281/zenodo.10342034. GitHub: https://github.com/taltendorf/Target-Sequence-Analysis-Tool-TSAT-.

SUPPORTING MATERIAL

Supporting material can be found online at https://doi.org/10.1016/j.bpr.2024.100166.

AUTHOR CONTRIBUTIONS

T.A. wrote the manuscript and developed the software. D.W. and J.M. supervised the project and revised the manuscript. J.M. tested the software.

ACKNOWLEDGMENTS

We sincerely thank Dr. Pauline Philippen, Dr. Marc Sevenich, and Dr. Karoline Santur-Dabow for their input and ideas during the planning phase of the project.

DECLARATION OF INTERESTS

The authors declare no competing interests.

REFERENCES

1. Smith, G. P. 1985. Filamentous fusion phage: novel expression vectors that display cloned antigens on the virion surface. *Science.* 228:1315–1317.
2. Kugler, J., J. Zantow, ..., M. Hust. 2013. Oligopeptide m13 phage display in pathogen research. *Viruses.* 5:2531–2545.
3. Wiesehan, K., and D. Willbold. 2003. Mirror-image phage display: aiming at the mirror. *Chembiochem.* 4:811–815.
4. Wiesehan, K., K. Buder, ..., D. Willbold. 2003. Selection of D-amino-acid peptides that bind to Alzheimer's disease amyloid peptide abeta1-42 by mirror image phage display. *Chembiochem.* 4:748–753.
5. Asar, M. C., A. Franco, and M. Soendergaard. 2020. Phage Display Selection, Identification, and Characterization of Novel Pancreatic Cancer Targeting Peptides. *Biomolecules.* 10:714.
6. Bussolati, B., C. Grange, ..., G. Camussi. 2007. Targeting of human renal tumor-derived endothelial cells with peptides obtained by phage display. *J. Mol. Med.* 85:897–906.
7. Li, C., N. Gao, ..., Y. Hou. 2017. Screening and identification of a specific peptide binding to cervical cancer cells from a phage-displayed peptide library. *Biotechnol. Lett.* 39:1463–1469.
8. Hou, L., D. Zhu, ..., X. Meng. 2018. Identification of a specific peptide binding to colon cancer cells from a phage-displayed peptide library. *Br. J. Cancer.* 118:79–87.
9. Yang, X., F. Zhang, ..., X. Su. 2016. A new non-muscle-invasive bladder tumor-homing peptide identified by phage display in vivo. *Oncol. Rep.* 36:79–89.
10. Leivo, J., M. Vehniainen, and U. Lamminmaki. 2020. Phage Display Selection of an Anti-Idiotype-Antibody with Broad-Specificity to Deoxynivalenol Mycotoxins. *Toxins.* 13.
11. Gallo, E., A. Kelil, ..., S. S. Sidhu. 2020. *In situ* antibody phage display yields optimal inhibitors of integrin $\alpha 11/\beta 1$. *mAbs.* 12:1717265.

12. Shin, Y. W., K. H. Chang, …, S. H. Kim. 2019. Selection of Vaccinia Virus-Neutralizing Antibody from a Phage-Display Human-Antibody Library. *J. Microbiol. Biotechnol.* 29:651–657.
13. Davidson, T. A., S. J. McGoldrick, and D. H. Kohn. 2020. Phage Display to Augment Biomaterial Function. *Int. J. Mol. Sci.* 21:5994.
14. Derda, R., S. K. Y. Tang, …, M. R. Jafari. 2011. Diversity of phage-displayed libraries of peptides during panning and amplification. *Molecules.* 16:1776–1803.
15. Santur, K., E. Reinartz, …, D. Willbold. 2021. Ligand-Induced Stabilization of the Native Human Superoxide Dismutase 1. *ACS Chem. Neurosci.* 12:2520–2528.
16. Kolkwitz, P. E., J. Mohrluder, and D. Willbold. 2022. Inhibition of Polyglutamine Misfolding with D-Enantiomeric Peptides Identified by Mirror Image Phage Display Selection. *Biomolecules.* 12.
17. Altendorf, T., I. Gering, …, D. Willbold. 2023. Stabilization of Monomeric Tau Protein by All D-Enantiomeric Peptide Ligands as Therapeutic Strategy for Alzheimer's Disease and Other Tauopathies. *Int. J. Mol. Sci.* 24:2161.
18. Sevenich, M., E. Thul, …, D. Willbold. 2022. Phage Display-Derived Compounds Displace hACE2 from Its Complex with SARS-CoV-2 Spike Protein. *Biomedicines.* 10:441.

Biophysical Reports, Volume *4*

Supplemental information

TSAT: Efficient evaluation software for NGS data of phage/mirror-image phage display selections

Tim Altendorf, Jeannine Mohrlüder, and Dieter Willbold

Target Sequence Analysis Tool (TSAT)

User Guide

Contents

Source code & Software 2

Objective of TSAT 2

How to use 3

Startup 3

Database creation 3

Translation direction 4

Framing region input 4

Input user and selection 7

Data insertion 7

Start data extraction and translation 8

Filtering of processed data 8

Q&A 9

Source code & Software

The Source code for TSAT can be found on Github:

https://github.com/taltendorf/Target-Sequence-Analysis-Tool-TSAT-

Alternatively, the source code can be found under the following DOI:

https://zenodo.org/doi/10.5281/zenodo.10342034

A released executable version of TSAT can be downloaded here:

https://github.com/taltendorf/Target-Sequence-Analysis-Tool-TSAT-/releases/tag/v1.0

Objective of TSAT

TSAT is a computational tool for the rapid analysis of NGS data obtained from Phage display and Mirror Image Phage display selections. It is able to extract desired genetic information, translate the information from DNA into protein and in an optional second step filter the obtained information for sequences with a higher probability to bind targets while excluding sequences that may not be target specific due to unspecific binding, amplification advantages or a bias in the library distribution. It was developed using the modules Tkinter, Bio, base64, re, collections, sqlite3, time, datetime, sys, threading and was written in Python version 3.6.5.

Prerequisite

TSAT accepts data in the form of FASTQ files encoded with UTF-8. To directly investigate the processed information a secondary program to visualize databases will be needed (e.g., db-browser for sqlite). Extraction of sequence information and translation into protein can be done without secondary programms. For the filtering of unwanted sequences as well as generation of different scores TSAT needs at least one library file, one target selection as well as one empty selection of the same round as input data.

How to use

Upon start TSAT needs information input from the user to function correctly. Following is a step-by-step guide on how to fill in all the information.

Startup

TSAT can be started by running the executable or source code with a python interpreter. After successful startup the user is able to see the following Window.

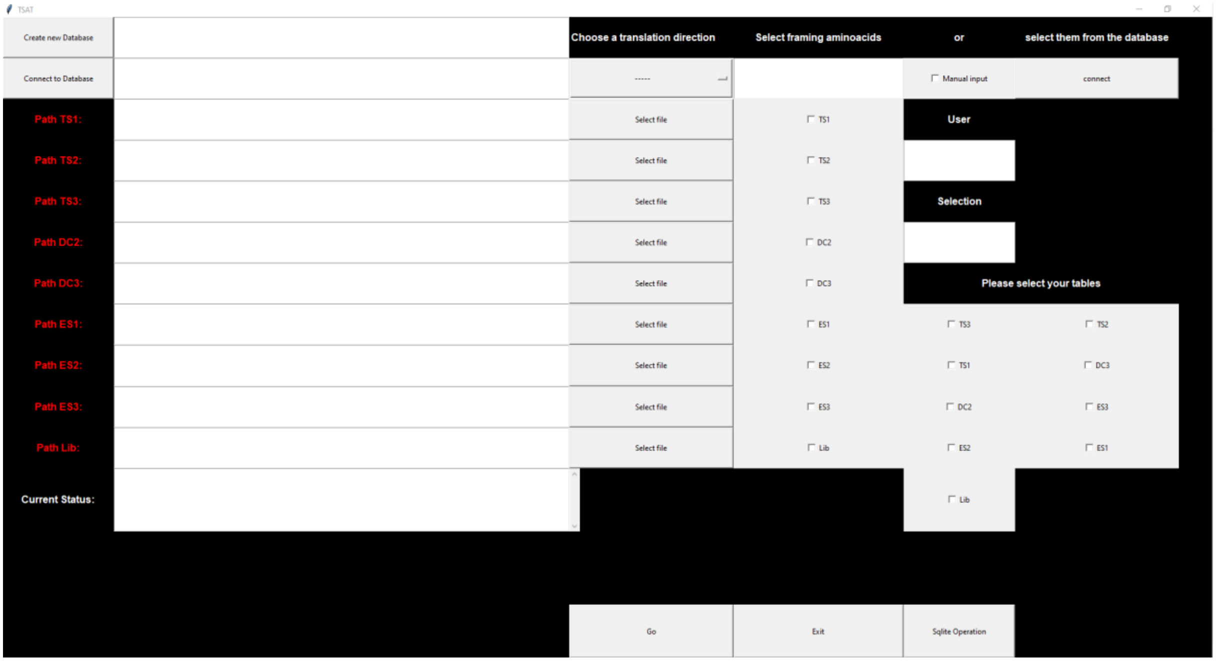

Database creation

In a first step the user needs to create a new database or connect to an existing one. It is recommended to create a new database for every run of TSAT to avoid loss or corruption of data. By clicking the “create new database” button the user is able to create and name a new database file (.db). Alternatively, the user can connect to an already existing database with the “Connect to Database” button.

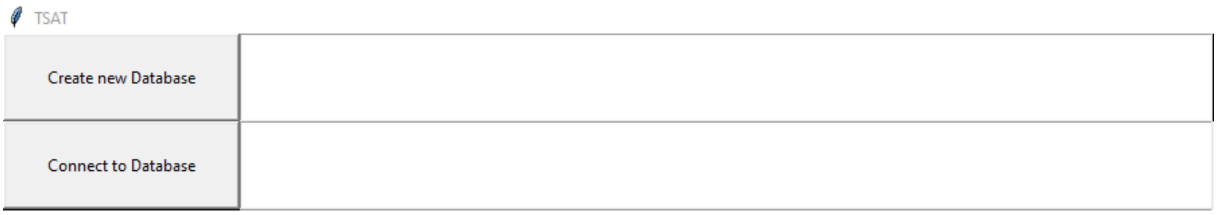

After creation of a new database, it is important to connect to the new database.

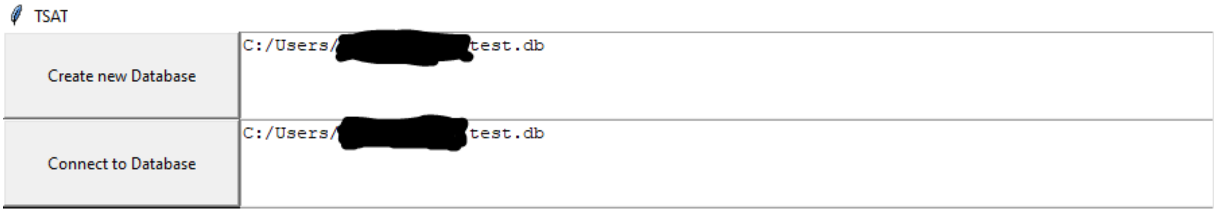

Translation direction

Here the user has to choose one of four possibilities for how the translation of excised DNA should be performed. Depending on how the phage library is set up the possibilities are Forward, Reverse, Forward complement and Reverse complement. This is done by choosing one of for options under the "Choose a translation direction" button. For the test data set the forward direction should be used.

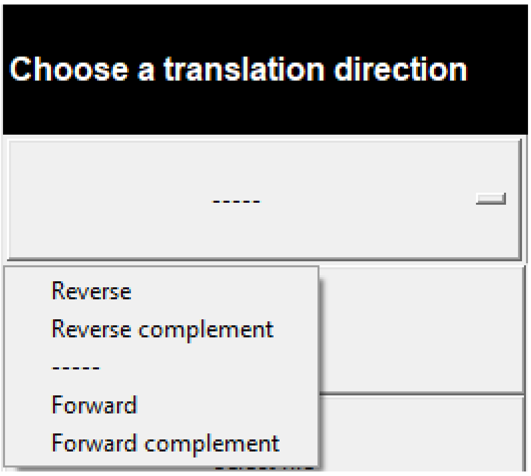

Framing region input

This step is of utmost importance as it provides TSAT with the information which random region is the region of interest .TSAT identifies desired nucleotide information via a regular expression function. This identification is dependent on framing regions surrounding the desired nucleotide information as they are part of the search pattern given to the function. For this you have two possibilities. The first one is to manually input the required information into TSAT. This option can be useful if you have a dataset that you only need to process once. For this you have to check the box "Manual Input" and insert the framing regions into the text box under "Select framing amino acids". Note that you have to give a defined amino acid composition before the randomized region and after it. The randomized region is displayed by (.+?)

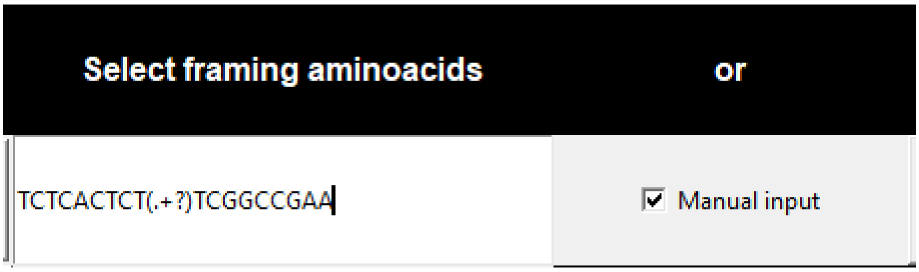

Alternatively, you can save your framing regions in a database and connect TSAT to this database. This is especially useful if you have many selections with the same randomized region, as it allows you to input the framing regions in a reduced amount of time with no spelling errors. In order to use this feature, you need to create a fresh database. This can be done clicking the "Create new Database" button in the top left corner of TSAT. After that you can connect to this database via the "Connect" button in the top right corner of TSAT. If you created a new database without any framing regions and connected to it two new buttons will appear below the "Connect" button.

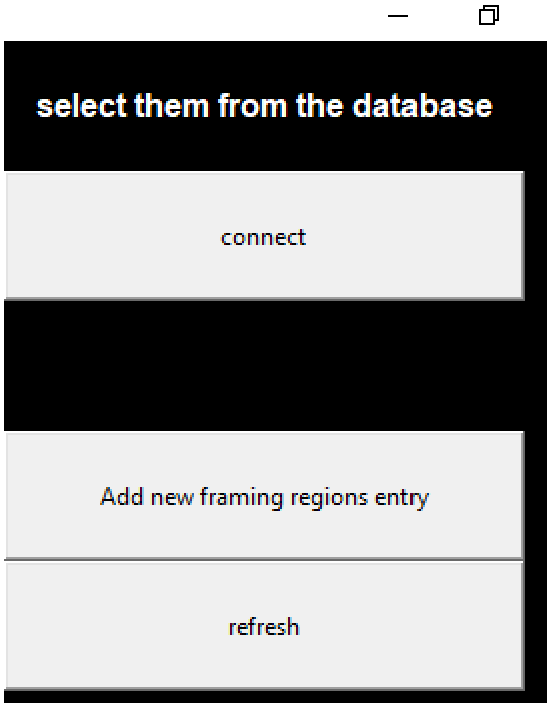

The next step is to add the wanted framing regions into the newly created database. This is done by clicking the button "Add new framing regions entry". By clicking this button, a new window will open where you can insert the needed Information.

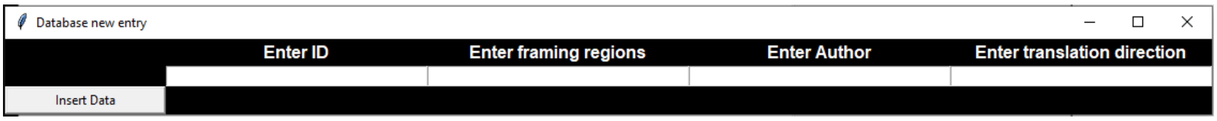

Here you need to give this framing region entry a name. This name will later be displayed in TSAT so that you can differentiate between different framing regions. Under framing regions, you have to enter the information that TSAT needs to find the desired randomized region. This is the same information you would have to enter in the manual mode. For this example, the framing regions that will be added are TCTCACTCT(.+?)TCGGCCGAA with (.+?) standing for the unknown randomized region. Afterwards you can add an author name which will only be saved in the database and not be displayed. Finally, you can enter a translation direction. At this moment the entry is also only saved in the database. It does not replace the drop-down selection within the main TSAT software. With the "Insert Data" button the

information is transferred to the database and saved within. Be sure that you have inserted everything correctly as you cannot change the data with TSAT. After this you can use the “refresh” button to update you connection with the Database. A new dropdown menu should appear in which you can select the entry you just created.

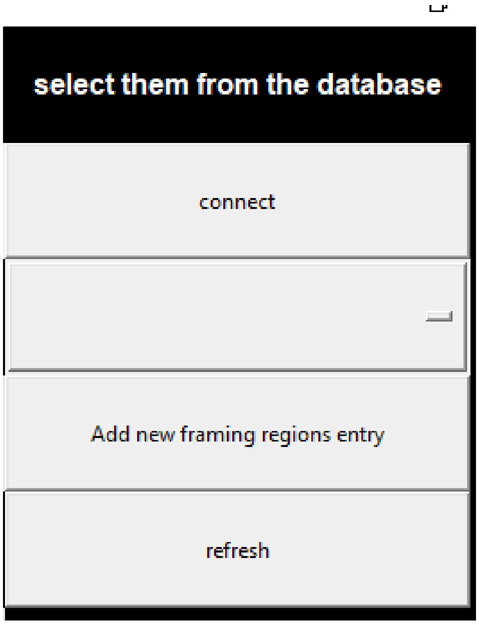

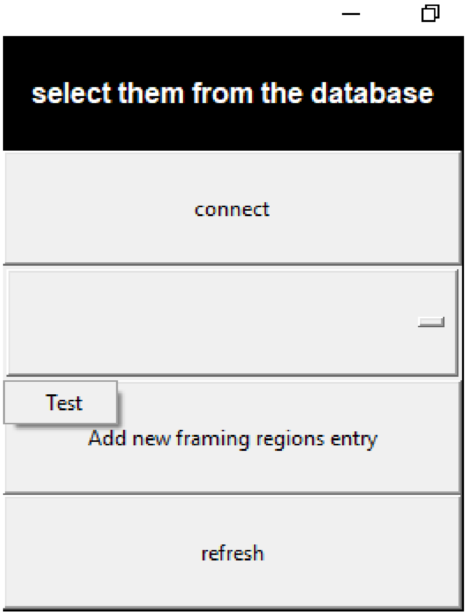

A test database containing the framing regions for the example dataset is provided on TSAT’s Github-page in the test folder. It is named framing regions.

Input user and selection

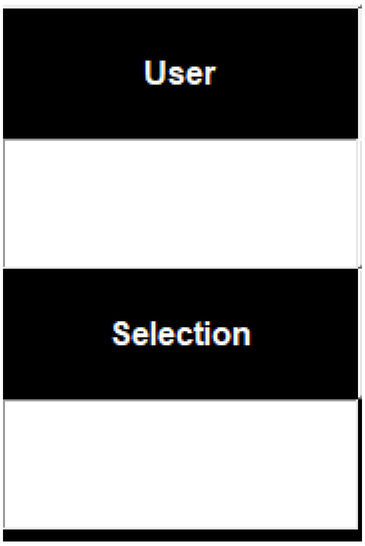

Here you can insert a user and a name for the selection. Both will be saved in the database. This is optional and TSAT will leave these areas blank if no entry was given.

Data insertion

The data that TSAT will analyze must be provided by the user using the “select file” button beside the textbox of “Path TS1” to “Lib”. The user is asked to provide a FASTQ file coded in UTF-8. Only one file can be given per path.

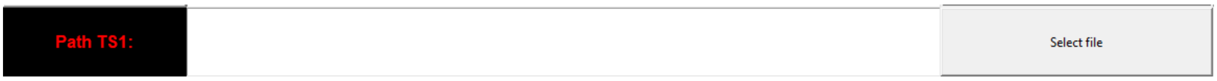

If the user wants to use the optional filtering it is important that the corresponding file is inserted into each path. TS stands for Target selection. This selection should be done against the target and is divided into 1-3 according to the increasing selection rounds. ES stands for empty selection. This selection is not against the target and should be done in parallel to the Target selection. It is used to identify sequences that are unlikely to be associated with the target but are present due unspecific binding. Again, this is divided into 1-3 according to the selection round. DC stands for Direct control. This selection was conducted against the target in the previous round and is subsequently performed without a target. DC selections are divided into 2 and 3 as the first round has to be the TS1 round. The library file is the final path option and represents the phage library state before a selection was performed.

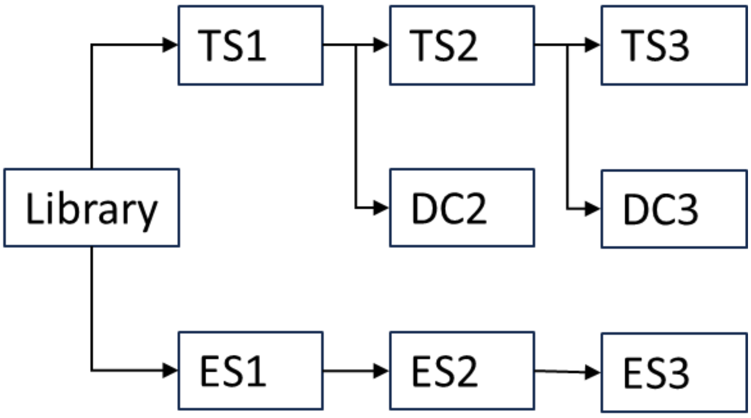

This file is used to identify sequences that are elevated in their frequency due to an amplification advantage or bias of the library. After file selection the path to the file is presented in the window. In case of a mix up it is possible to change the selected file by selecting another. After the files have been selected the user has to check which files were provided by marking the checkboxes to the right of the select file buttons.

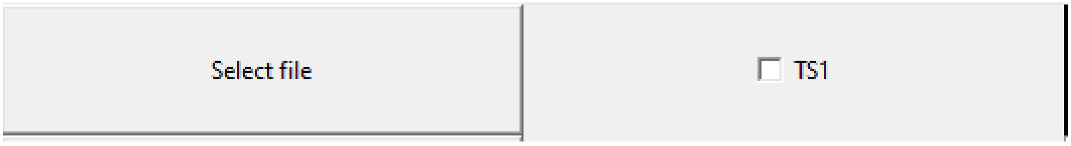

This is vital as TSAT won't operate on files that were not confirmed here. A user can use this feature to include or exclude a certain selection round for a second analysis without having to re-enter general Information. If a second analysis is performed, it is recommended to create a new database and connect to this newly created database. TSAT does not overwrite information already present in the database.

Start data extraction and translation

After all the required information has been entered and selected the operation may be started by using the "Go" button at the bottom of the program. TSAT will analyze the provided information and provide an overview over the progress in the current status box. An example of the displayed information would be the number of sequences that were found in a certain file. The textbox will also display the information when the processing has finished.

Filtering of processed data

The second functionality of TSAT is to filter the processed data in order to identify sequences that have a higher probability of being target specific and to exclude sequences that are thought to have a high frequency due to amplification advantage, starting bias or non-specific binding. To use this feature, one can either connect to a database containing at least one target selection, one empty selection and a library file or directly use the feature after finishing an extraction a translation with at least the mentioned files. To start the filtering, check the boxes corresponding to the files that are contained in the connected database under "please select your tables". The filtering is started by pressing the "Sqlite Operation" button at the bottom of TSAT. The filtering may take a while depending on the operating system. During the filtering TSAT will create two new scores to present two key features of every

sequence, the empty and enrichment score. The enrichment score represents the frequency of a sequence during its last target selection round divided by the frequency of the same sequence in the library.

$$Enrichment\ score = \frac{frequency\ of\ a\ sequence\ in\ TS3}{frequency\ of\ the\ same\ sequence\ in\ library}$$

This score is used to identify sequences that are only present as a result of a biased sequence distribution within the used phage library. The empty score represents the frequency of a sequence during its last target selection round divided by the frequency of the same sequence in the last empty selection round.

$$Empty\ score = \frac{frequency\ of\ a\ sequence\ in\ TS3}{frequency\ of\ the\ same\ sequence\ in\ ES3}$$

This score is used to identify sequences that are present as a result of binding to other components of the selection setup (e.g., plate, selection medium or blocking agent). TSAT excludes sequences that are smaller than 8 amino acids. Additionally, every sequence is analyzed for its enrichment from target round to target round as well as compared to the empty selection and direct control.

$$TS3 \geq ES3\ \&\ TS3 \geq DC3\ \&\ TS3 \geq TS2\ \&\ TS2 \geq ES2\ \&\ TS2 \geq DC2\ \&\ TS2 \geq TS1\ \&\ TS1 \geq ES1\&\ TS1 \geq Library$$

Sequences that do not increase from one target round to the next are excluded. Sequences that have a higher frequency in the corresponding empty selection and sequences whose frequency increases in a direct control compared to the previous target selection are removed. A new file is created after the filtering is completed. The user can decide where the file should be saved and might rename it. The file will be saved as a .fasta file. All sequences that were not discarded are saved in FASTA format. Sequences are sorted in descending order according to their respective Empty score. The empty score will be displayed in the .fasta file. In order to compare the enrichment scores a software to visualize the contents of a SQlite database is needed.

Q&A

Q: I see a star shape in my protein code, what is that?

A: The star shape is used if a stop codon was translated. This might happen when you use strains that substitute a certain stop codon with another amino Acid (e.g., amber suppressor).

Q: How can I review the translated data that is stored in the database?

A: TSAT cannot display the translated data that is stored in the database. For this please use a different software that is able access and display such data. As an example, you could use DB browser for SQLite

Q: How does TSAT handle sequences that are not present in the empty selection or library?

A: In this case the sequence will be handled as if it was present 0.5 times in the selection or library.

Q: Why do I need the library file for filtering?

A: The library is used to check the state before a selection occurred. In the filtering it serves as a “target selection 0”.

Q: Why do I see the same sequence twice or more often?

A: This happens when there are multiple codons that code for the same amino acid. TSAT orders at the level of DNA meaning those “duplicated” sequences all have a different genetic code.
